# Patient Compliance With Physical Therapy Following Orthopedic Surgery and Its Outcomes

**DOI:** 10.7759/cureus.37217

**Published:** 2023-04-06

**Authors:** Abdullah E Kattan, Hadi B AlHemsi, Ahmed M AlKhawashki, Faisal B AlFadel, Saad M Almoosa, Abdulmalik M Mokhtar, Bassam A Alasmari

**Affiliations:** 1 Department of Surgery, Division of Plastic Surgery, King Khalid University Hospital, Riyadh, SAU; 2 College of Medicine, King Saud University Medical City, Riyadh, SAU

**Keywords:** saudi arabia, questionnaire, compliance, physiotherapy, orthopedic surgery

## Abstract

Background and Objectives: Patient compliance is a major concern for the efficacy of physiotherapy amongst those that undergo orthopedic surgery. The substantial number of people who are non-compliant makes this an imperative issue to address. Our objectives were to quantify the percentage of patient compliance for physiotherapy after their surgery, to measure the association between compliance and the status of health, mobility, and pain, and to identify the causes of non-compliance.

Methods: A cross-sectional study was conducted on post-orthopedic surgery patients attending physical therapy sessions at King Khalid University Hospital in Riyadh, Kingdom of Saudi Arabia, over a one-year period. The sample size of 359 was calculated and selected using simple random sampling. Our questionnaire was developed by adopting questions from two previously validated studies.

Results: The majority of the participants (n=194; 54%) were male. One hundred and ninety-three (53.8%) participants had a diploma or higher. The age group 18-35 was found to be significantly associated with skipping physiotherapy sessions when they started to feel well (P= 0.016) and skipping due to other responsibilities (P=0.002). Single people skip physiotherapy when they start to feel well (P=0.023), due to other responsibilities (P=0.028), and due to poor timing (P=0.049). Self-reported compliance to physical therapy after surgery was 231 (64.3%). Patient status showed overall improvement.

Conclusion: There is a significant percentage of non-compliance and the patient's age, gender, marital status, and level of education play a role in the causes of non-compliance. In addition, the patient’s status (health, pain, and mobility) is better in those who are compliant than in those who are not.

## Introduction

Orthopedic surgery or orthopedics is concerned with conditions involving the musculoskeletal system. Orthopedic surgeons specialize in certain areas of the body such as the hip, knees, and elbows [1]. On the contrary, physical therapists or physiotherapists plan and implement rehabilitative programs that improve or restore motor functions, maximize movement ability, and treat or prevent physical challenges associated with injuries or diseases. Some of the benefits of physiotherapy, especially in targeted sessions, include reducing or eliminating pain, improving mobility, and managing heart and lung diseases among other conditions [2].

Disability is part of human nature and whether it is attributed to the wear and tear that occurs with aging or otherwise, the impairment associated with it will affect nearly everyone at some point in life, temporarily or permanently [3]. Physiotherapy has the highest evidence of effectiveness in avoiding recurrence and chronic disability. However, the effectiveness of physiotherapy can be directly related to the patients' adherence to the plan [4]. Given such facts, physical therapy is a means to decrease the burden of disability and find solutions for chronic diseases such as rheumatoid arthritis [5]. Physical therapy services reduce hospital length of stay for adults [6].

Compliance is a means to an end, an approach to maintaining or improving health as well as managing symptoms and signs of disease. Compliance, also known as adherence, is a complex behavioral process strongly influenced by the environments in which patients live, healthcare providers practice, and healthcare systems deliver care [7]. Patient non-compliance is believed to be a major concern for the efficacy of physiotherapy amongst those that undergo orthopedic surgery. Mainly, three factors are related to noncompliance and they include the barriers patients encounter, the lack of positive feedback, and the degree of helplessness [8].

The substantial number of people who are non-compliant makes it an imperative issue to address, and this is why we aim to identify the percentage of these patients in King Khalid University Hospital (KKUH), Riyadh, Saudi Arabia. Because of the importance of physiotherapy as mentioned, our objectives were to quantify the percentage of patient compliance with physiotherapy after their orthopedic surgery, to measure the association between compliance and the status of health, mobility, and pain, and to identify the causes of non-compliance to physiotherapy within six months after surgery.

We hypothesize that the percentage of compliance among postoperative orthopedic patients who require physiotherapy within six months of their surgery is 60%. We also hypothesize that in this group, those who comply with their physiotherapy sessions show an overall improvement in their health compared to those who do not comply. We also hypothesize that the lack of determination and engagement in other life responsibilities for some patients is a major factor for non-compliance.

## Materials and methods

This cross-sectional study was conducted in Riyadh, Kingdom of Saudi Arabia at KKUH over 12 months. The data collection was conducted on a daily basis starting from September 21, 2021, to October 4, 2021, using the help of phone calls. The study was approved by the Institutional Review Board of King Saud University in Riyadh, Kingdom of Saudi Arabia, on August 2, 2021, (reference number: 21/0640/IRB; approval number: E-21-6167) and consent was obtained from the targeted population.

The daily data collection period was divided into two smaller time frames, the first interval being from 9 am to 12 pm and the second interval being from 1 pm to 4 pm. Our population of interest was orthopedic surgery patients undergoing physical therapy at KKUH in Riyadh, Kingdom of Saudi Arabia. Using a population of 1428, which was collected through a list provided by KKUH's IT department, then taking 50% of the population to estimate the number of orthopedic surgery patients in the latter half of the year 2020 and a confidence level of 95% with a confidence limit of 5%, the sample size calculated via Epi Info™ (Centers for Disease Control and Prevention, Atlanta, Georgia, United States) was 250. The final sample size was adjusted to 359 after accounting for a 30% questionnaire non-response rate, and we selected the participants from our population using a simple random sampling technique.

Before being surveyed, participants were assessed for fulfilling the inclusion and exclusion criteria. The inclusion criteria were the following: Arabic or English-speaking patients who are aged 18 and above who have undergone orthopedic surgery at KKUH, Riyadh, Saudi Arabia, and have undergone or are still undergoing sessions of physical therapy. The exclusion criteria were: patients requiring medications only (without the need for physiotherapy), pregnant patients, patients who underwent orthopedic surgery over a year ago, and patients who started physiotherapy for the first time after six months of undergoing their orthopedic surgery.

Participants were asked if they were willing to participate in our study knowing that none of the collected data could possibly identify a participant, such as a name or a phone number. By accepting the terms and conditions, consent was fully acquired, ensuring their anonymity. No incentives or rewards were given to participants. Study variables included age, gender, level of education, marital status, and occupation. The outcome variable was the percentage of compliance.

Questionnaire

After completing an in-depth review of the literature regarding this topic of interest and conducting a thorough search of articles on both PubMed and Google Scholar, we adopted and modified a number of questions from two questionnaires in order to craft our very own questionnaire provided in the appendix. The first is from a validated patient compliance questionnaire conducted via a face-to-face interview seeking information about general rehabilitation adherence in physical therapy patients visiting clinics for musculoskeletal disorders [2]. The second is a telephone-based questionnaire seeking the adherence and effectiveness of rehabilitation in acute ankle sprains [9]. The crafted online-administered questionnaire had five sections and was available in two language versions, Arabic and English.

In both versions, the first section included demographic data questions. Participants were divided into six age groups (18-25, 26-35, 36-45, 46-55, 56-65, and 66+), which were then collapsed into three (18-35, 36-55, >=56), gender classification was into two (male or female), level of education was categorized into five groups (high school diploma, bachelor’s degree, master’s degree/post-graduate degree, doctorate, and other), which were also collapsed into three (illiterate, pre-highschool/highschool, diploma/bachelor’s/post-graduate). Marital status was divided into five categories (single, married, widow/widower, separated, other); this was also collapsed into three (single, married, widow/widower/divorced). Finally, occupation was also divided into five categories (student, employed, unemployed, housewife/househusband, retired).

The second section included knowledge-based questions in order to check the level of information and knowledge the participants had. All questions had one correct answer based on the currently accepted literature in orthopedics, physiotherapy, and rehabilitation. Participants had to choose one of the following: yes, no, I don’t know.

In the third section, the questions were made to reflect the preferences of patients in practicing physical therapy after orthopedic surgery. Answers were categorized as: disagree, agree, neutral.

The fourth section included descriptive questions regarding the patients' experiences before, during, and after the course of treatment. Answering was based on choosing one of the following per period (terrible, bad, mediocre, good, excellent)

The fifth and final section included questions measuring compliance and adherence in physical therapy post-orthopedic surgery. Compliance status was measured by the way participants answered those questions, which was choosing one of the following: never, rarely, sometimes, often, always. There was no missing data as all participants answered all questions. We ensured participants' privacy by only collecting the specific data needed for the research and we didn't collect any information that identified the participants or led to them.

Data analysis

The data were analyzed using IBM SPSS Statistics for Windows, Version 26.0 (Released 2019; IBM Corp., Armonk, New York, United States). Frequency percentages were calculated for categorical variables. The Chi-square test was used to measure the association between categorical variables. A p-value of <= 0.05 was used to report the statistical significance of the results. Compliance was calculated by scoring 15 questions with a max score of 46; one question about how often they attended their sessions was scored as follows: always and often =4, sometimes, rarely, and never =0. The other 14 questions were about common causes of skipping sessions and were scored as follows: always and often =0, sometimes = 1, rarely and never =3. Then all results were summed to find the average score of 37, using the (IF) command to automatically place those who scored 0 in the first question directly as non-compliant, while the rest were considered compliant if they scored >=37. Health, mobility, and pain status were recorded, where 0 symbolizes bad status and 1 symbolizes good status, taking the mean of all responses at each point (before, during, after) and visualizing it in a graph to show progression.

## Results

A total of 359 participants fulfilled all inclusion criteria and were included in the study. The majority of the participants (n=194; 54%) were male. In terms of age, 179 (49.9%) participants were between 18 to 35 years old. One hundred and eighty-two (50.7%) were married. One hundred and ninety-three (53.8%) participants had a diploma, bachelor’s, or postgraduate degree, and 158 (44%) participants were employed. The distribution of other sociodemographic characteristics is given in Table 1.

**Table 1:** Distribution of sociodemographic characteristics of orthopedic patients undergoing physiotherapy (n=359)

Variable	Value	N (%)
Gender	Male	194 (54)
	Female	165 (46)
Age (in years)	18-35	179 (49.9)
	36-55	83 (23.1)
	>=56	97 (27)
Marital status	Single	138 (38.4)
	Married	182 (50.7)
	Widow/Widower/Divorced	39 (10.9)
Education	Illiterate	33 (9.2)
	Pre-high school/high school	133 (37)
	Diploma/Bachelor’s/Post-graduate	193 (53.8)
Occupation	Student	51 (14.2)
	Employed	158 (44)
	Unemployed	54 (15)
	Housewife/Househusband	96 (26.7)

Self-reported compliance to physical therapy sessions after undergoing orthopedic surgery was 231 (64.3%). All patients began the physiotherapy session within six months of their surgery. Two hundred and thrity-nine (66.6%) patients performed physiotherapy in the hospital, 86 (24%) at home, and the rest practiced at various different places. Three hundred and forty-nine (97.2%) patients believed that physiotherapy is used to promote, maintain and restore health, 347 (96.7%) found it necessary after surgery, 352 (98.1%) believed that compliance to physiotherapy improved mobility, while only 275 (76.6%) patients said that post-surgical complications can be managed with physiotherapy. A total of 375 (91.1%) agreed that they must complete the ordered physiotherapy course, and 335 (93.3%) said it was important to perform a variety of exercises in general. Three hundred and twenty (89.1%) believed that they must be supervised during their sessions.

Most of the patients (n=99; 27.6%) skipped sessions due to other responsibilities while skipping due to poorly timed sessions comes second with 91 (25.3%) patients answering yes, and skipping when they feel unwell is another prominent cause (n=89; 24.8%). The least common cause is skipping due to emotional reasons with only 22 (6.1%) patients answering yes. Seventy-eight (21.7%) skipped sessions when they started feeling well, 72 (20.1%) skipped when an assigned physiotherapist was not available, 69 (19.2%) skipped when they felt burned out, 58 (16.2%) skipped due to financial reasons, 55 (15.3%) of patients skipped when they felt a lack of outcome, 52 (14.5%) skipped due to lack of available companion to be there with them, 46 (12.8%) skipped due to therapy-inflicted pain, 35 (9.7%) skipped when they felt stressed, and only 33 (9.2%) skipped if their assigned physiotherapist was available. 

The univariate analysis shown in Table 2 is for the association of sociodemographic variables with the common causes of skipping physiotherapy sessions. The age group of 18-35 years was found to be significantly associated with skipping sessions when they started to feel well (P= 0.016) and skipping due to other responsibilities (P=0.002) compared to the other age groups. Similarly, those who were single were more likely to skip when they started to feel well (P=0.023), due to other responsibilities (P=0.028), and due to poor timing (P=0.049). Males were more likely to skip due to other responsibilities (P=0.044) than females. Patients with a diploma degree or higher were also more likely to skip due to other responsibilities (P=0.013) and poor timing (P=0.049). Occupation status was found to be insignificant with all common causes, and the rest of the causes were found to be insignificant with all variables.

**Table 2:** Association of causes of non-compliance with sociodemographic characteristics

Variable	Skipping sessions when starting to feel well				Skipping sessions due to other responsibilities				Skipping due to poor time			
	Yes (%)	No (%)	x²	p-value	Yes (%)	No (%)	x²	p-value	Yes (%)	no (%)	x²	p-value
Age												
18-35 years	50 (27.9)	129 (72.1)	8.241	0.016	64 (35.8)	115(64.2)	12.870	0.002	53 (29.6)	126 (70.4)	3.699	0.157
36-55 years	14 (16.9)	69 (83.1)			19 (22.9)	64 (77.1)			16 (19.3)	67 (80.7)		
56+ years	14 (14.4)	83 (85.6)			16 (16.5)	81 (83.5)			22 (22.7)	75 (77.3)		
Gender												
Male	49 (25.3)	145 (74.7)	3.094	0.079	62 (32)	132 (68)	4.059	0.044	51 (26.3)	143 (73.7)	0.167	0.657
Female	29 (17.6)	136 (82.4)			37 (22.4)	128 (77.6)			40 (24.2)	125 (75.8)		
Marital status												
Single	41 (29.7)	97 (70.3)	8.797	0.012	49 (35.5)	89 (64.5)	7.165	0.028	43 (31.2)	95 (68.8)	6.05	0.049
Married	29 (15.9)	153 (84.1)			42 (23.1)	140 (76.9)			36 (19.8)	146 (80.2)		
Widow/widower/divorced	8 (20.5)	31 (79.5)			8 (20.5)	31 (79.5)			12 (30.8)	27 (69.2)		
Level of education												
Illiterate	4 (12.1)	29 (87.9)	2.732	0.255	3 (9.1)	30 (90.9)	8.635	0.013	6 (18.2)	27 (81.8)	6.04	0.049
Pre-high school/High school	27 (20.3)	106 (79.7)			33 (24.8)	100 (75.2)			26 (19.5)	107 (80.5)		
Diploma/Bachelor’s/Post-graduate	47 (24.4)	146 (75.6)			63 (32.6)	130 (67.4)			59 (30.6)	134 (69.4)		
Occupation												
Student	12 (23.5)	39 (76.5)	2.207	0.531	20 (39.2)	31 (60.8)	6.793	0.079	18 (35.3)	33 (64.7)	3.184	0.364
Employed	36 (22.8)	122 (77.2)			47 (29.7)	111 (70.3)			38 (24.1)	120 (75.9)		
Unemployed	14 (25.9)	40 (74.1)			12 (22.2)	42 (77.8)			12 (22.2)	42 (77.8)		
Housewife/Househusband	16 (16.7)	80 (83.3)			20 (20.8)	76 (79.2)			23 (24)	73 (76)		

**Figure 1:**
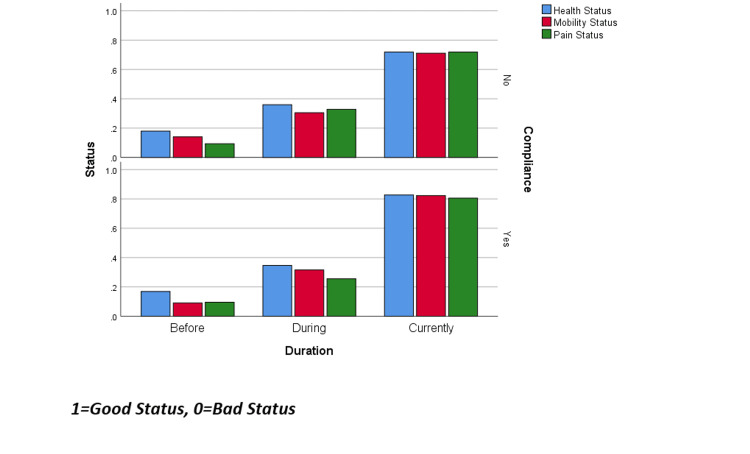
Progression of health, mobility, and pain from before starting the physiotherapy session till the time of data collection

Figure 1 depicts slightly more improvement in health, mobility, and pain in compliant patients in comparison to non-compliant patients.

## Discussion

We concluded that the level of compliance among the participants was 64%. The level of compliance was found to be consistent with similar conclusions found in a previous study conducted by Di Fabio et al., in which the rate of compliance ranged from 85% to 89% in patients suffering from orthopedic diseases [10]. Other studies, local and international, however, stated high levels of non-compliance. Ludwig and Adams, in their study, reported that 44% of patients completed their scheduled course of physiotherapy [11]. Recent reports also showed that non-compliance to rehabilitation among patients with lower back pain may be as high as 50% [12,13]. While our study yielded a relatively lower non-compliance percentage of 36% compared to the aforementioned studies, it is still considered to be high.

The reasons for non-compliance vary. Booysen found that the level of compliance to medication was affected by severe pain influencing sleep and movement [14]. In a systemic review of oral medication compliance, Mathes et al. concluded that a financial reason, i.e. higher medication costs, seems to have a negative effect on compliance [15].

We found that patients' age affected the association between the level of compliance and skipping physiotherapy when starting to feel well and skipping due to other responsibilities, especially in the age group of 18-35 years. This is probably attributed to age being moderately correlated with the initial intensity of pain, reflecting that older patients had higher pain scores at their first physiotherapy visit. Research conducted in the Riyadh region of Saudi Arabia showed that age was significantly correlated with adherence, indicating that older patients were more adherent to physiotherapy [4]. In another study, seven systematic reviews were included and showed that there is a tendency that higher or middle age is associated with higher adherence. But in more than half of the reviews, the effect was unclear [15].

The marital status of patients affected the association between the level of compliance and skipping physiotherapy when starting to feel well, skipping due to other responsibilities, and skipping due to poor time, especially patients who are single. This is probably attributed to married people having greater family support that encourages them to continue to adhere to their physical therapy sessions. However, the Riyadh-based study done by Al-Eisa showed that marital status was not associated with adherence [4].

Patients' gender affected the association between the level of compliance and skipping physiotherapy due to other responsibilities, especially male patients. This is probably attributed to cultural habits of the place where this study was conducted. Conversely, in an experimental study conducted to assess the level of adherence between groups of males and females, the study found that the male group was more adherent to the exercise program and spent more time exercising compared to the female group [16].

Patients' level of education affected the association between the level of compliance and skipping physiotherapy due to other responsibilities, and skipping due to poor time, especially patients with an education level of diploma/bachelor’s/post-graduate. While unexpected, it could be due to higher educated patients bearing more responsibilities and thus having less time to attend physical therapy sessions. On the other hand, a study showed that higher education and employment status seem to have a positive effect on adherence [15]. Another study found that most patients who had basic education appeared adherent as compared to uneducated patients [2]. In a study done by Adams, educated patients were observed to be more adherent as compared to uneducated ones [17]. It is worth mentioning that educated patients make informed decisions about their health. Such patients have better health literacy and are able to understand their treatment needs. Thus, there is a significant likelihood that educated patients would know the importance of complying and adhering to treatment options.

When comparing compliant and non-compliant patients in terms of health, mobility, and pain status, this study’s results show that at the start of the physiotherapy treatment, discrepancies in terms of health, mobility, and pain status were negligible, whereas over the course of the treatment, these discrepancies begin to widen, and so later on in the course, health status, mobility status, and pain status are all slightly better in compliant patients compared to non-compliant patients. This is attributed to the fact that all patients achieved maximum compliance at the start of the program and this was thus followed by maximum effectiveness from the physical therapy sessions, which caused the majority of the patients to become less compliant with the sessions, which led to the discrepancy created between the two groups in terms of health, mobility, and pain status.

In a study conducted to understand reasons for compliance and non-compliance with a home-based exercise regimen by patients with osteoarthritis of the knee, results showed that the initial period had the same effectiveness in terms of pain and mobility between the patients. After a follow-up period of five months, by which time the majority of patients were probably partially compliant at best, the effect of the physiotherapy became diluted by those who were not compliant [18].

Strengths and limitations

A major strength can be attributed to the collection of data from a variety of different age groups from the targeted population, Including diverse age groups showed that non-compliance is indeed affected by age. By using our questionnaire, we were able to collect data on all variables at once.

A major limitation can be related to the the lack of an apparent gold standard in measuring compliance in modern literature. An issue in terms of our use of a questionnaire as a data collection method is that it may have resulted in a risk of misclassification and alteration of the real-life situation due to recall bias and response bias. Selection bias may be a factor to limit the ability to generalize the data to the larger population, as we have focused on collecting data from the patients of a single university hospital. Also, the study was conducted over a relatively short period of 12 months. This may limit the generalizability of the findings, as patient behaviors and attitudes toward physical therapy may vary over time. The last limitation results from the difficulty of taking into consideration the many confounders that are present such as the severity of the injury or surgery.

## Conclusions

Our results suggest a significant level of non-compliance to physiotherapy in patients who underwent orthopedic surgery. It appears that each patient’s age, gender, marital status, and level of education are associated with the compliance level to the physiotherapy treatment sessions. It is also shown that the higher the level of compliance the better the results in terms of health, mobility, and pain. 

Further studies are needed to examine compliance in its different conditions. In addition to being directed toward developing techniques and strategic methods to improve the level of compliance with physiotherapy, building a good relationship with patients, explaining the treatment plan, giving clear instructions, and showing how the exercises will help patients reach their goals are all points that would improve compliance immensely.

## Figures and Tables

**Figure 2:**
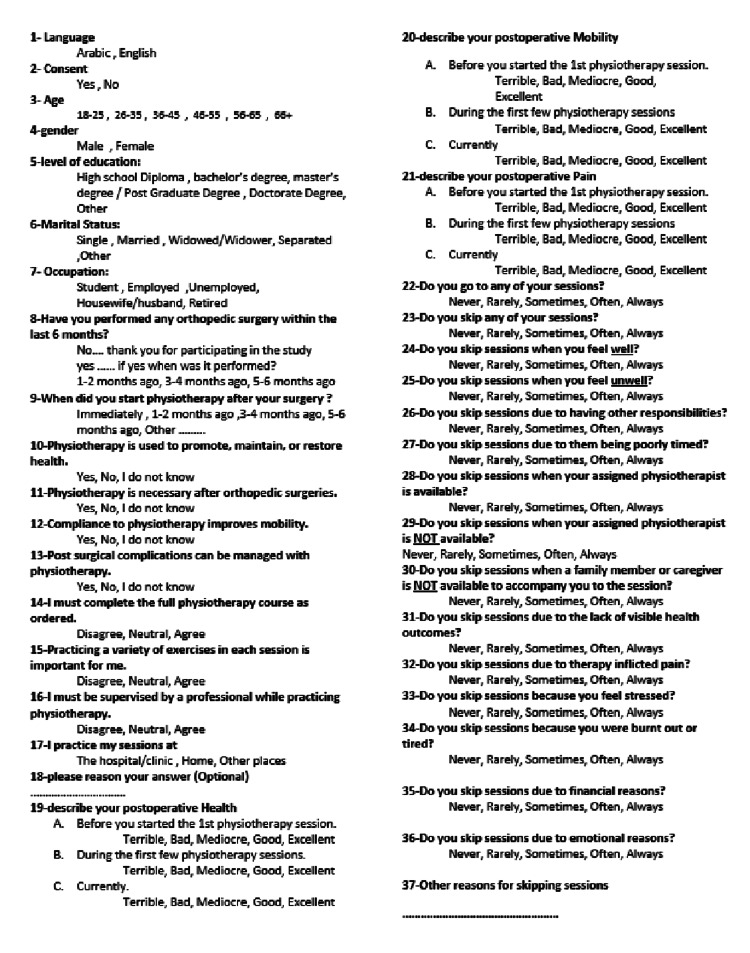
English version of the questionnaire

**Figure 3:**
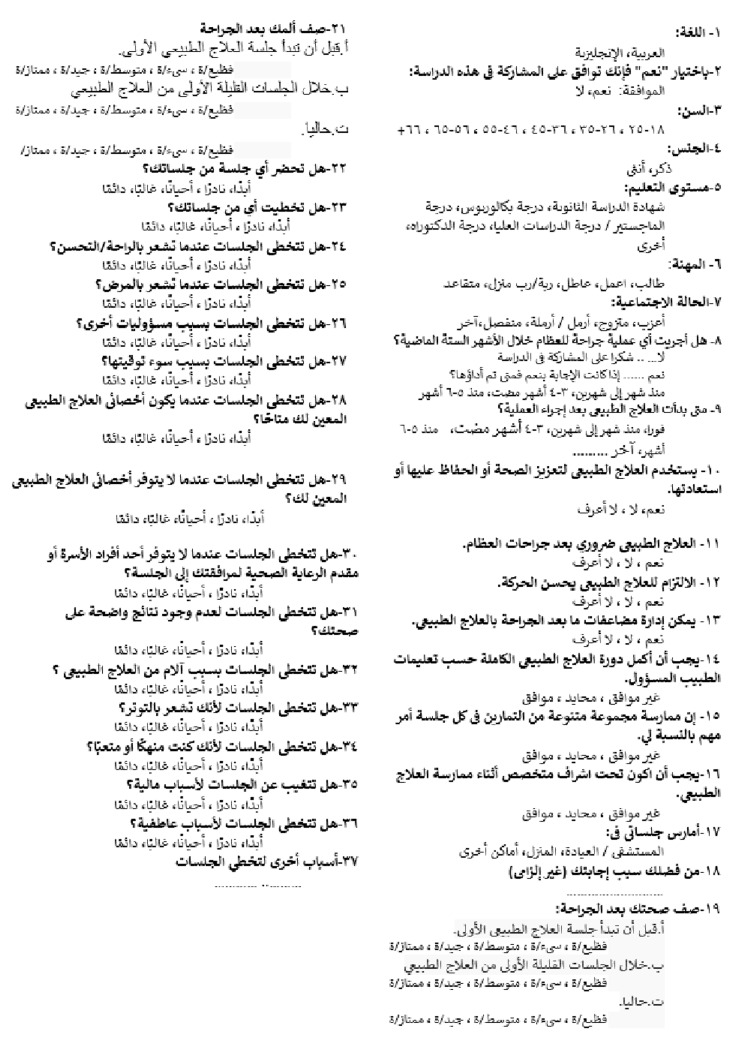
Arabic version of the questionnaire
